# Eutrophication in Poyang Lake (Eastern China) over the Last 300 Years in Response to Changes in Climate and Lake Biomass

**DOI:** 10.1371/journal.pone.0169319

**Published:** 2017-01-03

**Authors:** Mengna Liao, Ge Yu, Ya Guo

**Affiliations:** 1 University of Chinese Academy of Science, Beijing, PR China; 2 College of Chemistry and Life Sciences, Zhejiang Normal University, Jinhua, PR China; 3 State Key Laboratory of Lake Science and Environment, Nanjing Institute of Geography and Limnology, Chinese Academy of Sciences, Nanjing, PR China; University of Brighton, UNITED KINGDOM

## Abstract

Poyang Lake is suffering from persistent eutrophication, which is degrading the local ecosystem. A better understanding of the mechanisms that drive eutrophication in lake systems is essential to fight the ongoing deterioration. In this study, hydraulic residence time (HRT) was used to evaluate Poyang Lake’s trophic state. A hydrology and ecosystem forced model was constructed to simulate long-term changes in algae and aquatic plant biomass and total phosphorous (TP). A comparison analysis revealed that between 1812 and 1828 (i.e., a consistent-change stage), climate and hydrology were the main driving forces, while algae and aquatic plant biomass contributed only 20.9% to the trophic changes in Poyang Lake. However, between 1844 and 1860 the biomass predominated contributing 63.6%. This could be attributed to nutrient absorption by algae and aquatic plants. A correlation analysis of the water TP and algae and aquatic plant biomass revealed a strong positive relationship. However, the algae and aquatic plant growth rate tended to decline after the biomass reached half of the maximum. This research reconstructs the long-term trophic evolution of Poyang Lake and provides a better understanding of the relationship between climatic and hydrological changes and lake ecosystems.

## Introduction

Lake eutrophication is strongly linked to nutrient changes over a long time [1,2]. Shallow lakes are much more vulnerable than deep water ecosystems because they have a low capacity for contaminants or nutrient loads (e.g., [3,4]). For this reason, shallow lakes can easily change from a “clear state” to a “turbid state” [5]. During such a transition, the lake morphology and hydrodynamics are important factors in a shallow lake ecosystem [6,7]. Eutrophication in shallow lakes has become a global issue (e.g., [8,9]).

Under natural conditions, lake eutrophication generally occurs on a centennial timescale [10,11]. Previous studies have provided some perspective for understanding eutrophication mechanisms. For example, May [12] proposed a preliminary theory regarding the multiplicity of stable states in a lake ecosystem. More recently, Scheffer and Carpenter [13] reached a similar conclusion. They noted that gradual changes in temperature or other factors might have little effect until they reach a threshold at which a large shift occurs that is difficult to reverse. Other studies of eutrophication processes discussed several other factors, such as hydrological conditions and lake morphology (e.g., [4,14,15]), competition between different ecological communities (e.g., [16]), water-sediment exchange and nutrient input (e.g., [17,18]), and climate change (e.g., [19]). However, lake eutrophication is not yet fully understood because such phenomena have normally been investigated using records for a short timescale.

Dynamic ecosystem models have been applied to explore eutrophication mechanisms from different perspectives, focusing on resource competitions between different ecological communities (e.g., [20,21]), chemical impacts on aquatic ecosystems (e.g., [22]), interactions between different communities and the surrounding environment (e.g., [23,24]), the critical nutrient load or the threshold for lake trophic transitions (e.g., [25,26]), and the effects of hydrological and climatic factors (e.g., [27]). Such models have mainly focused on explaining the dynamic relationships between different communities and between communities and the surrounding environment. Similar models quickly developed after the Lotka-Volterra model was proposed in the 1920s [28,29]. This model predicted that predators thrive when there is plentiful prey but ultimately outstrip their food supply and decline. As the predator population decreases, the prey population increases. These dynamics continue in a cycle of growth and decline. The Lotka-Volterra model served as a starting point for the development of several other models, such as the ecological population competition model, the mutual benefit and collaboration model, and a model that introduced a time-delay term [30]. However, these models have difficulty describing phenomena that occur over a longtime period, such as the abrupt collapse of the African forest steppe vegetation into dry desert [13,31]. Although Claussen et al. [32] and Liu et al. [33] successfully developed dynamic climate models with vegetation feedback to simulate long-term changes in African vegetation, few researchers have applied a dynamic ecosystem model to simulate the long-term evolution of a lake ecosystem.

The shallow lakes in the middle and lower reaches of the Yangtze River have received considerable attention due to their accelerated eutrophication. Poyang Lake is the largest freshwater lake in China and is located along the south bank of the middle reach of the Yangtze River. Until the end of the 20th century, the water in Poyang Lake was of relatively good quality. It started deteriorating at the end of that century [11,34] and is currently in transition to a state of eutrophication [35]. Although the catchment area has been thoroughly studied with respect to hydrology, meteorology, and ecology (e.g., [36–40]), most of these studies lacked a long-term perspective regarding nutrient change and acceleration of the eutrophication process. Therefore, they missed an important factor that may have explained the eutrophication mechanism in this lake [41]. Key lake eutrophication issues are closely related to water quality, which is linked to climatic and hydrological conditions, lake morphology and their interactions with biology [7,42]. Thus, identifying the key factors that trigger abrupt change must rely on studies in physical models of long-term trophic changes and the previously mentioned factors.

To elucidate Poyang Lake’s trophic evolution and eutrophication trend, we used two approaches in this paper: (1) sediment records and nutrient proxies from lake cores to reconstruct long-term processes, and (2) models constructed to investigate hydraulic residence and ecosystem-nutrient dynamics in the lake. The former approach qualitatively described the historical trophic changes, and the latter approach quantitatively simulated the nutrient balance and biological feedback. Moreover, the data allowed comparison and verification of the modeling results.

## Study Site

**Fig 1:**
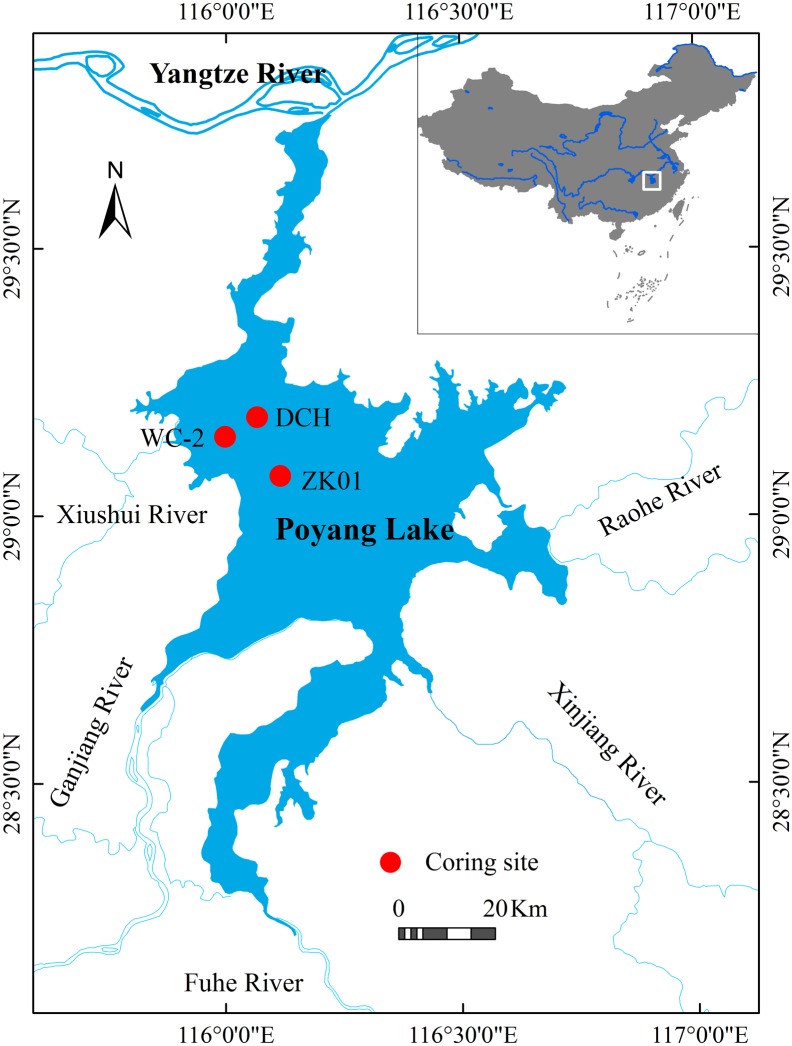
Location of Poyang Lake and the coring sites.

Poyang Lake is situated along the south bank of the Yangtze River (28°24′-29°46′ E, 115°49′-116°46′ N; Fig 1). It is China’s largest freshwater lake and has distinct morphological characteristics during flood and drought periods. During the dry season, the lake covers a relatively small area of 146 km^2^ and has a water volume of 4.5×10^8^ m^3^. During the flood season, these dimensions greatly change and the lake can reach up to 3000 km^2^, with an approximate water volume of 150×10^8^ m^3^. The lake catchment area is 16.2×10^4^ km^2^, and the recharge coefficient is 55 during the flood season. Poyang Lake’s multiple-year mean high and low water levels are respectively 20.69 m and 9.82 m above sea level (a.s.l.). The Gajiang, Fuhe, Xinjiang, Raohe and Xiushui Rivers are five main sources that recharge Poyang Lake. The only natural outlet is in Hukou County, north of the lake, where the lake water runs into the Yangtze River. During the flood season, the Yangtze River serves as a water source for the lake, but during the remainder of the year, the lake discharges water into the river [43]. This means that the hydraulic residence time (on average 10–60 days from 1953–1984) [43] was shorter compared to other shallow lakes in the mid-lower Yangtze basin. The lake region is dominated by a subtropical monsoon climate with an annual temperature of 16.5–17.8°C, precipitation of 1570 mm and evaporation of 1236 mm [10].

Phytoplankton and aquatic plants are the most important primary producers in Poyang Lake. The minimum distribution density of phytoplankton generally occurs in February and is approximately 27×10^4^ cells/L, while the density reaches a maximum of 355×10^4^ cells/L in September and October [43]. The annual mean phytoplankton density is approximately 47.6×10^4^ cells/L [43]. The monthly concentration range of Chlorophyll-*a*/L in 1988 ranged from 0.469 μg to 2.171 μg, with an average of 1.272 μg [43]. A high concentration usually occurs in the summer and autumn, while a low concentration occurs in the spring and winter [43]. The dominant species of phytoplankton changes seasonally. Summer is dominated by cyanobacteria, while during the spring, diatoms dominate the lake. Late autumn and winter are dominated by dinoflagellates. The primary aquatic macrophytes in Poyang Lake consist of hygrophytes and emerged and submerged plants. The dominant species include *Carex*, *Phragmites* and *Potamogeton*. Their contributions to the total biomass of hygrophyte, emerged and submerged communities were approximately 34.7%, 29.7%, and 15.8%, respectively (in 1988) [43].

## Materials and Methods

No specific permission was required for the field study. The lake is not privately owned, and the field study did not involve endangered or protected species.

### Sediment records and reconstruction methods

In November 2012, a 1-m sediment core (WC-2) was taken from the western lakeshore of Poyang Lake (29°11′53″ N, 115°59′46″ E, 13.20 m a.s.l.).

The chronology of the top 50 cm of the core was established by using the ^210^Pb/^137^Cs dating method (S1 Fig). The analysis was carried out in the Stake Key Laboratory of Lake Science and Environment at the Nanjing Institute of Geography and Limnology, CAS. A constant rate of supply (CRS) model [44] was used to establish the chronology and calculate the sedimentation rates. Because the half-life of ^210^Pb is 22.3 years, it can only be used to date recent sediments (0–150 years) [44]. Therefore, an alternative approach was used to estimate the ages of the lower part of the sediments. Considering that the lithology of core WC-2 was homogenous, mainly composed of silt and clay and had no sediment gaps along the core profile, we used the average sedimentation rate of the upper part (0.321 cm/a) to extrapolate the ages of the lower-part sediment. The results showed that the bottom layer was approximately 300 years old.

Core WC-2 was subsampled at intervals of 1 cm for diatom identification and analysis. Diatom samples were treated with HCl and H_2_O_2_ to remove carbonates and organic material [45]. Two replicate subsamples were potentially available from each sample. Diatom valves were enumerated on each of two slides prepared from each sample. The diatoms were identified under oil immersion microscopy at 1000× magnification with an Olympus microscope (BX51). The diatoms were identified according to diatom manuals [46–49]. A diatom percentage diagram (S2 Fig) was drawn using TILIA and TILIAGRAPH software [50]. Because the diatoms in the sediment were not well preserved due to the strong hydrodynamics of Poyang Lake, only 100 valves could be identified and counted per sample.

Because of the close proximity of core ZK01 (29°1′58″ N, 116°8′26″ E, 13.24 m a.s.l.) and core DCH (29°9′00″ N, 115°5′24″ E, 12.2 m a.s.l.) to core WC-2, we speculated that they would have similar sedimentation characteristics. Accordingly, we used proxy data from cores ZK01 and DCH to compare with records from core WC-2. The long-term series of aquatic pollen concentrations from core ZK01 and the total organic carbon (TOC) from core DCH have been published [51,52]. Because the lake sediment TOC (‰) has been considered as a proxy of organic productivity [53–55], we used it as a proxy of the lake primary biomass, including the aquatic plant and phytoplankton biomass. The aquatic pollen concentration anomaly (%) (the percentage variations of aquatic pollen concentration relative to the mean aquatic pollen concentration of all samples taken from the lake) was used as a proxy to indicate changes in the aquatic plant biomass. Cores WC-2, ZK01, and DCH were taken at 13.20 m, 13.24 m and 12.2 m a.s.l., respectively, which fell well within the range of lake level fluctuations (9.8–20.7 m a.s.l.). Hence, all three cores should record changes in the water level and sediment environment. During the last 30 years, the emphasis of palaeolimnology has shifted from a predominantly qualitative, descriptive subject to a quantitative, analytical science [56]. The most significant development in quantitative palaeolimnology has been the creation of many modern calibration datasets of biotic assemblages and associated environmental data [56]. Calibration data consist of two parts. One is a modern training set that consists of units or samples of modern biological data and associated environmental data, and the other is the fossil set that consists of units or samples of fossil biological data [57,58]. The former are used to establish a transfer function that expresses the relationship between ecological components (such as diatoms, chironomids, and cledocerae) and targeted environmental factors (TP, TN, water temperature, conductivity, and pH, for example). The palaeoenvironmental factor can subsequently be reconstructed based on the function established and the fossil set. Although the use of diatom-inferred (DI) transfer functions is controversial [59], they have been successfully applied to palaeolimnology studies in many lakes (e.g., [60–62]), including those in the middle-lower reaches of the Yangtze River (e.g., [38–40,63]). In this study, we used the fossil diatom data derived from core WC-2 and an existing diatom-inferred TP (DI-TP) transfer function to estimate the TP concentrations in Poyang Lake for the past 300 years. The DI-TP transfer function was developed from a set of 45 lakes in the middle-lower reaches of the Yangtze River [37]. The function was established by several types of weighted averaging (WA) models and weighted averaging partial least squares (WA-PLS) models [37]. The DI-TPs were validated with observed TPs. Yang et al. [37] suggested that a refined inverse-deshrinking WA model was one of the best and had the lowest root mean squared error of prediction (RMSEP = 0.124) and the highest coefficient of prediction (R^2^_jack_ = 0.819). The final DI-TP transfer function can be expressed as [37]:

$${\text{log}}_{\text{10}}TP=a+bx_{i}.$$ (1)

where *x*_*i*_ represents the initial diatom-inferred TP (μg/L) for the *i*th sample, *a* = -2.1295, and *b* = 2.0944. The quantitative TP reconstruction for core WC-2 was performed with C2 version 1.5 software [59].

### Modern climatic, hydrological, and ecological data

The climatic database from 14 meteorological stations in the Poyang Lake catchment (National Meteorological Information Center, China Meteorological Administration) spans a time interval between 1880 and 2008. However, the data are not continuous because of wars and economic crises during the period that resulted in a lack of information before 1950. To obtain a longer time series, the observed precipitation and temperature data were interpolated by using records from Wuhan Station from 1880, Jiujiang Station from 1885, Nanchang Station from 1885 and Jingdezhen Station from 1929. These records indicate that only Wuhan Station monitored precipitation and temperature from 1880–1884, so we used these data as precipitation and temperature data for Poyang Lake for 1880–1884. The precipitation and temperature between 1885 and 1928 could be interpolated by using data from Wuhan, Jiujiang and Nanchang stations, while information for 1929–1950 could be interpolated by using data from all four stations. The interpolation was conducted with the Inverse Distance Weighting (IDW) method [64]. Additionally, monthly potential evaporation was calculated using the Thornthwaite method [65]. Hydrological observations (1954–2008) were sourced from the Yangtze Water Resource Commission, Water Resources Ministry. Water discharge data for Poyang Lake were obtained from the Hukou Hydrological Station.

The lake volume, surface area, catchment area, and lake depth were obtained for 1980–1990 [10]. Lake biological data (including phytoplankton, aquatic plant and fish biomass) were collected from multiple-line investigations in the 1980s [43,66–68] and individual ecological studies [35,69–72] (S1 Table). Briefly, the phytoplankton included Chlorophyta, Bacillariophyta, Cyanobacteria, Chrysophyta, Euglenophyta, Xanthophyta, Pyrroptata and Cryptophyta. The aquatic plant consisted of hygrophytes, emergent plants and submerged plants. The fish community was mainly composed of carp, crucian, bighead, and chub, which are the most important fish in Poyang Lake. Based on the data collected, the ratio of the biomass of algae to aquatic plants to fish was estimated to be 100:145:1 (S1 Table).

### Historical climate and hydrology data

**Fig 2:**
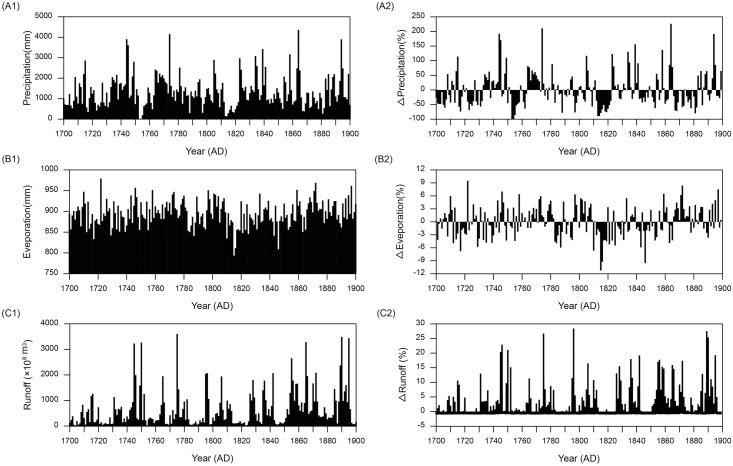
Results of meteorological and hydrological simulations (1700–1899). (A1) ECHAM5-simulated monthly precipitation (mm); (A2) Simulated precipitation anomaly (%); (B1) ECHAM5-simulated monthly potential evaporation (mm); (B2) Simulated potential evaporation anomaly (%); (C1) WATLAC-simulated monthly runoff (m^3^) of Poyang Lake; (C2) Simulated runoff anomaly (%).

Due to the lack of meteorological data prior to 1880, we used the output of the global climate model ECHAM5 [73] for the period 1700–1899. The climate simulation was conducted under boundary conditions that included changes in solar irradiation, tephra, and greenhouse gases [74]. The temperature and precipitation in the Poyang region (30.00°N, 117.500°E ~ 25.313°N, 112.500°E) from 1700–1899 (Fig 2A1 and 2A2) were obtained using downscaling methods [75]. Potential evaporation (Fig 2B1 and 2B2) for this period was calculated by using the Thornthwaite method [65]. The percentage variation of temperature and precipitation relative to the mean values of precipitation and evaporation series were defined as precipitation and evaporation anomalies in this study.

Discharges from Poyang Lake from 1700–1899 were simulated by using the WATLAC distributed catchment-hydrology model [76], which has been previously applied to Poyang Lake catchment for simulating discharge and has been validated [76–79]. The validation results showed that the relative error between the modeled and observed annual discharges was less than 8% (1960–1989) [76–79] and 1.9%~12.3% (1991–1995) [76], and the Nash-Sutcliffe efficiency was in the range of 0.71~0.84 (2000–2008) [79], which suggests that the WATLAC model is applicable to Poyang Lake. Precipitation and potential evapotranspiration are the main driving forces in this model, so the simulated precipitation and potential evaporation were used as the main boundary conditions for 1700–1899. The results of simulated discharges are shown in Fig 2C1 and 2C2 and the definition of the discharge anomaly is similar to that of the precipitation and evaporation anomalies.

### Simulation methods

#### Model 1: Hydraulic residence time

The hydraulic residence time (HRT) is a measure of the average length of time that a compound (e.g. water) remains in a storage unit (e.g., a lake, pond, or ocean) [64]. It can be expressed as follow [64]:

HRT=V/Q, (2)

Q=P+R−E. (3)

where *V*, *Q*, *P*, *E* and *R* represent the lake volume (m^3^), discharge (m^3^/a), water surface precipitation (mm/month), water surface evaporation (×10^−3^ mm/month), and the catchment-derived runoff (m^3^/a). For an inter-annual timescale perspective, we converted *P* and *E* into units of mm/a. Additionally, we adopted the *HRT* anomaly (Δ*HRT*), which is the percentage variation (%) relative to the mean value of *HRT* series, to reflect variations or fluctuations.

#### Model 2: Lake ecology-nutrient dynamics

Primary producers in lakes mainly include planktonic microalgae and aquatic vascular plants. To depict the major long term changes in their biomass, our simplified model was focused on the two terminals of the ecosystem food chains. The fish community acts as a predator and was placed at the top, while algae and aquatic plant communities serve as prey and primary producers, and were placed at the bottom.

Determining the ecological relationship between the planktonic microalgae community (algae community) and the aquatic vascular plant community (plant community) is the key to constructing an ecological dynamic model. A competitive relationship exists between the algae and plant communities, because both are aquatic primary producers and compete for light and nutrition in the water and sediment to sustain life. In addition, algal excretion can inhibit the growth of aquatic plants, and vice versa [80,81]. On the other hand, the predator-prey relationship between the fish community and the algae and plant communities also plays an important role in the food chain. Therefore, competitive relationships between the algae and plant communities and their predator-prey relationships with the fish community constitute a two-terminal ecological structure.

Based on the Lotka-Volterra model [28,29], the total biomass of the three communities in a lake and their changes over time can be represented by the differential equations below. To simulate the seasonal and annual changes of biomass, a time function *g*(*t*) [30] that describes the changes in the biotic communities over different time scales was added to the equations.

$$\begin{array}{l} y_{1}{}^{\prime }=y_{1}\left(a_{1}+a_{2}y_{3}+a_{4}y_{1}+g_{1}\left(t\right)\right),\\ y_{2}{}^{\prime }=y_{2}\left(b_{1}+b_{3}y_{3}+b_{4}y_{2}+g_{2}\left(t\right)\right),\\ y_{3}{}^{\prime }=y_{3}\left(c_{1}+c_{2}y_{1}+c_{3}y_{2}+c_{4}y_{3}+g_{3}\left(t\right)\right). \end{array}$$ (4)

where *y*_*1*_, *y*_*2*_, and *y*_*3*_ represent the algae, plant and fish community biomass, respectively, *y’* refers to *dy(t)/dt*, *a*_*1*_, *a*_*2*_, and *a*_*4*_ are parameters of the algae community, *b*_*1*_, *b*_*3*_, and *b*_*4*_ are parameters of the aquatic plant community, and *c*_*1*_, *c*_*2*_, *c*_*3*_, and *c*_*4*_ are parameters of the fish communities. Parameters marked “1” are the intrinsic rate of increase. Those marked “2” and “3” are community interaction parameters. Parameters marked “4” are the ratio of the intrinsic rate of increase to their environmental capacity. The parameters were statistically determined and validated by the nonlinear optimization fitting method based on ecological investigations of Poyang Lake between 1980 and 2000 [82].

Nutrients are the limiting resource for phytoplankton. Previous studies have summarized many relationships that are practical for analyzing the effects of nutrients on phytoplankton growth and competition and the nutrient uptake and intake processes of algae [83]. These models were established based on the quantitative relationships between nutrients and algae growth. Even though different mathematical expressions have been used to described those relationships, those expressions share a basic format that can be written as:

$$N_{a}=rN_{c}/\left(k+N_{c}\right).$$ (5)

where *N*_*a*_ is the increment of the algae biomass, *r* is the maximum increment, *N*_*c*_ is the nutrient concentration, and *k* is the Michaelis-Menten constant. The units of *N*_*c*_ and *k* are mg/L.

Aquatic plants absorb nutrients primarily from the lake sediment. However, the relationship between the aquatic plants and nutrients remains uncertain [84,85]. Previous studies used a linear equation to describe the relationship between the nutrient concentration and the aquatic plant biomass (e.g., [86]):

$$N_{p}=mN_{c}+e.$$ (6)

where *N*_*p*_ is an increment of the aquatic plant biomass, *m* is the slope, *N*_*c*_ is the nutrient concentration (mg/L), and *e* is the intercept (e = 0 after differentiation). This equation can also be used to describe the relationship between the fish biomass and nutrients.

The effect that phytoplankton and aquatic plants have on nutrient uptake/intake (-*N*) and release/excretion (+*N*) must be considered when constructing an equilibrium relationship among different communities (i.e., the algae, aquatic plant, and fish communities in the present study). Designating *m*_*1*_, *m*_*2*_, and *m*_*3*_ the coefficients of the growth equations [Eqs (5) and (6)] for the algae, aquatic plant, and fish, Eq (4) can be expressed as:

$$\begin{array}{l} y_{1}{}^{\prime }=y_{1}\left(a_{1}+a_{2}y_{3}+a_{4}y_{1}+m_{1}N/\left(k_{1}+N\right)g_{1}\left(t\right)\right),\\ y_{2}{}^{\prime }=y_{2}\left(b_{1}+b_{3}y_{3}+b_{4}y_{2}+m_{2}Ng_{2}\left(t\right)\right),\\ y_{3}{}^{\prime }=y_{3}\left(c_{1}+c_{2}y_{1}+c_{3}y_{2}+c_{4}y_{3}+m_{3}Ng_{3}\left(t\right)\right). \end{array}$$ (7)

Nutrients accumulated in a lake (Δ*N*) can be described as the balance between nutrient input from the lake catchment (*N*_*catch*_) and the nutrients absorbed (-) or released (+) by the lake organisms (*N*_*bio*_):

$$\Delta N=N_{0}+N_{\text{catch}}-N_{bio}.$$ (8)

where *N*_*0*_ is the initial nutrient load. Δ*N* can be estimated by the product of inflow (*W*) and the nutrient concentration (*N*): Δ*N* = *W N*.

Designating *y*_4_ as lake nutrients, the differential equation for lake nutrient balance can be expressed as:

$$y_{4}{}^{\prime }=y_{4}\left(W_{0}+W\right)-y_{4}\left(d_{1}k_{1}/\left(1-y_{1}\right)y_{1}+d_{2}y_{2}+d_{3}y_{3}\right).$$ (9)

where *W*_*0*_ is the initial inflow, *W* is the variations of inflow, *d*_*1*_, *d*_*2*,_ and *d*_*3*_ represent the nutrient uptake rate of the algae, aquatic plant, and fish communities, respectively. By combining Eq (7) with Eq (9), the interaction and feedback between nutrients and biomass can be described as:

$$\begin{array}{l} y_{1}{}^{\prime }=y_{1}\left(a_{1}+a_{2}y_{3}+a_{4}y_{1}+m_{1}y_{4}/\left(k_{1}+y_{4}\right)g_{1}\left(t\right)\right),\\ y_{2}{}^{\prime }=y_{2}\left(b_{1}+b_{3}y_{3}+b_{4}y_{2}+m_{2}y_{4}g_{2}\left(t\right)\right),\\ y_{3}{}^{\prime }=y_{3}\left(c_{1}+c_{2}y_{1}+c_{3}y_{2}+c_{4}y_{3}+m_{3}y_{4}g_{3}\left(t\right)\right),\\ y_{4}{}^{\prime }=y_{4}\left(W_{0}+W-d_{1}k_{1}/\left(1-y_{1}\right)y_{1}-d_{2}y_{2}-d_{3}y_{3}\right). \end{array}$$ (10)

The units of *y*_*1*_, *y*_*2*_, *y*_*3*_, and *y*_*4*_ are t/month. In order to reflect the variations or fluctuations of the algae, aquatic plant, and fish biomass (*y*_*1*_, *y*_*2*_, *y*_*3*_) and the nutrient content (*y*_*4*_), we converted them into anomalies (%). The definition for anomalies in this paper is similar to that of Δ*HRT*.

## Results

### HRT changes in the past 300 years

**Fig 3:**
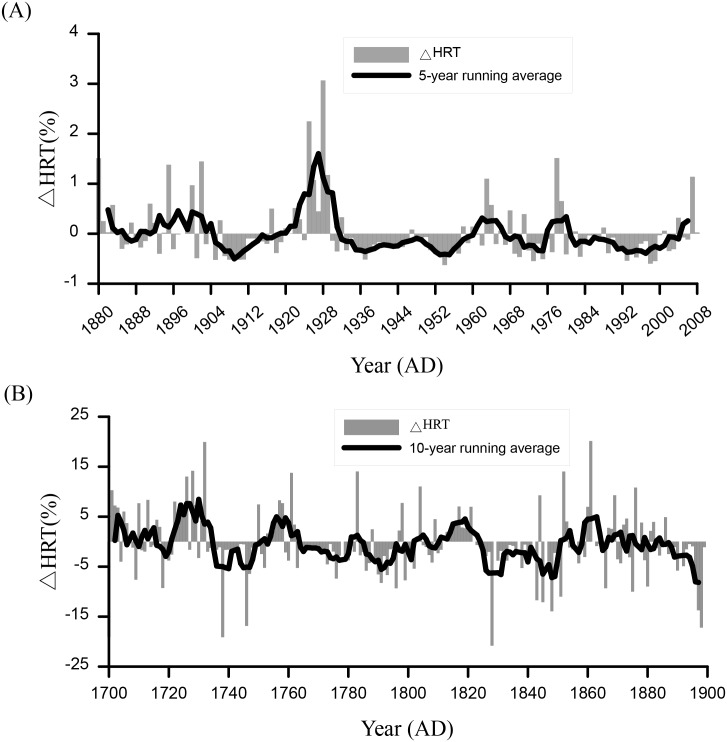
HRT anomaly (ΔΔHRT) in Model 1 and its change process. (A) ΔHRT calculated with observed meteorological and hydrological data for 1800–2008; (B) ΔHRT calculated with simulated meteorological and hydrological data for 1700–1899.

We simulated an HRT series between 1880 and 2008 based on Model 1 and the observed meteorological and hydrological data, and then converted it into ΔHRT. We used a 5-year running average of the ΔHRT series to reflect the change process (Fig 3A). We divided the series into five stages (1880–1901, 1902–1928, 1929–1950, 1951–2000, and 2000–2008) according to the process to analyze the variation trends. We used a linear trend analysis in this study to analyze the variation trends. The analysis results showed that: (1) From 1880–1901, the variation ranged -0.49% to +1.51%. The trend coefficient was approximately -0.010, showing a slight decline. (2) From 1902–1928, the ΔHRT changed intensely in a range of -0.52%~+3.07% with a trend coefficient of +0.052, which indicated an increasing trend. (3) From 1929–1950, ΔHRT was relatively stable, with variation ranging from -0.52% to +1.17%, and a slight decline was indicated by the trend coefficient of -0.013. (4) From 1951–2000, the ΔHRT changes remained in the range of -0.63%~+1.51%. Two small peaks occurred in 1964 and 1979. The trend coefficient (-0.003) showed a very small decrease. (5) From 2000–2008, the ΔHRT varied in the range of -0.34%~+1.14. The trend coefficient was +0.069, indicating an increasing trend.

An earlier reconstruction of the HRT (1700–1899) was conducted based on Model 1, the ECHAM5-simulated meteorological data and the WATLAC-simulated discharges. The resultant HRT series was also converted to ΔHRT. A 10-year running average of the ΔHRT series reflected frequent fluctuations (Fig 3B). Four stages (1700–1724, 1725–1764, 1765–1850, and 1851–1899) were considered to elucidate the change process. The results of linear trend analyses show that: (1) During the earliest period from 1700–1724, the ΔHRT ranged from -10.26% to +19.02%. The trend coefficient was +0.058, indicating an increasing trend during this period. (2) From 1725–1764, the ΔHRT ranged between -19.92% to +19.11%. The trend coefficient was -0.066, which indicated a decreasing trend. (3) From 1765–1850, the ΔHRT changes remained in a range of -14.03%~20.81%. A declining trend was indicated by a trend coefficient of -0.018. 4) Finally, from 1851–1899, the ΔHRT ranged from -20.12% to +17.19% and the trend coefficient of -0.162 indicated a declining trend.

### Nutrient and biomass changes in the past 300 years

**Fig 4:**
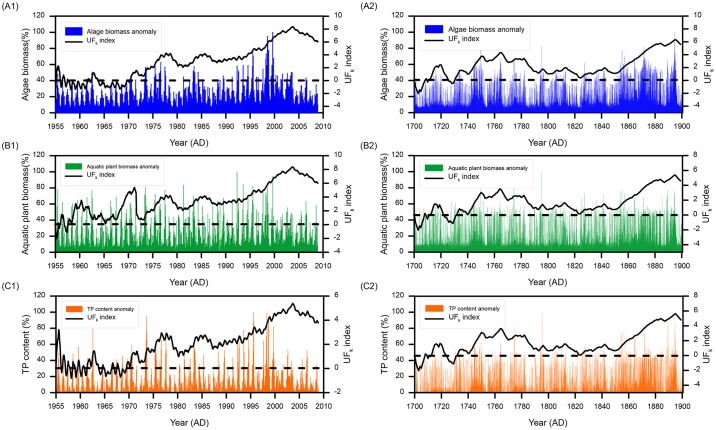
Simulations of Model 2 and Mann-Kendall trend analyses. Anomaly series of monthly algae biomass and the corresponding UF_k_ index from 1955–2008 (A1) and 1700–1899 (A2); Anomaly series of monthly aquatic plant biomass and the corresponding UF_k_ index from 1955–2008 (B1) and 1700–1899 (B2); Anomaly series of monthly TP content and the corresponding UF_k_ index from 1955–2008 (C1) and 1700–1899 (C2).

To calibrate and validate Model 2, we first ran a modern simulation for the period from 1955–2008 (Fig 4A1 and 4B1) and validated it by comparing the observed and simulated biomass data. Based on ecological investigations of Poyang Lake, the ratio of biomass of algae to aquatic plants and fish was estimated as 450:650:4.5 (S1 Table). The simulation results indicated a ratio of 480.5:676.9:4.09 [87]. The simulation errors were +6.8% for the algae biomass, +4.2% for the aquatic plant biomass, and -9.1% for the fish biomass. We considered a maximum error of less than 10% as acceptable.

We used the Mann-Kendall (MK) test [88,89] to perform trend analysis to detect the changes in algae and aquatic plant biomass and the TP content from 1955–2008. The UF_k_ index was calculated at a significance level α = 0.05. The results showed that the algae biomass anomaly series did not obviously change before 1970 (Fig 4A1). Afterward, it increased and fluctuated. Two small peaks occurred in 1978 (UF_k_ = 4.23) and 1985 (UF_k_ = 4.17). From 1987–1995, the biomass increased steadily. From 1996–2003, the increasing trend was obvious, and the maximum appeared in 2004 (UF_k_ = 8.38). Then the increasing trend subsided. The UF_k_ index of aquatic plant biomass implied frequent fluctuations before 1975 (Fig 4B1). Two declining trends occurred in 1955 (UF_k_ = -2.03) and 1957 (UF_k_ = -1.12). Strong increasing trends occurred from 1958–1960 (UF_k_ increased to 3.52) and 1967–1971 (UF_k_ increased to 5.40), followed by two declines in 1961–1967 (min UF_k_ = 0.73) and 1972–1974 (min UF_k_ = 0.78). The trend after 1975 was similar to that for the algae biomass. The overall changes in the TP content anomaly was also similar to that of the algae biomass, except that the period 1955–1956 showed an obviously increasing trend (max UF_k_ = 3.22, Fig 4C1).

Biomass and TP simulations for 1700–1899 also showed frequent fluctuations (Fig 4A2, 4B2 and 4C2). We used the MK test to analyze these trends as well. The UF_k_ index series of algae and aquatic plant biomass and TP content anomalies were very similar (Fig 4A2, 4B2 and 4C2). Two declining trends appeared in 1700–1707 and 1726–1730. The minimum UF_k_ indexes were -2.04 and -0.48, respectively. Significant increase occurred in 1716–1720, 1745–1787 and 1857–1899 AD, as indicated by an UF_k_ index >1.96. The UF_k_ index peaked (UK_k_ = 6.34) in 1896. From 1788–1856, no obvious changes were evident in the three anomaly series. The UF_k_ index is ranged from 0.38~1.94.

**Fig 5:**
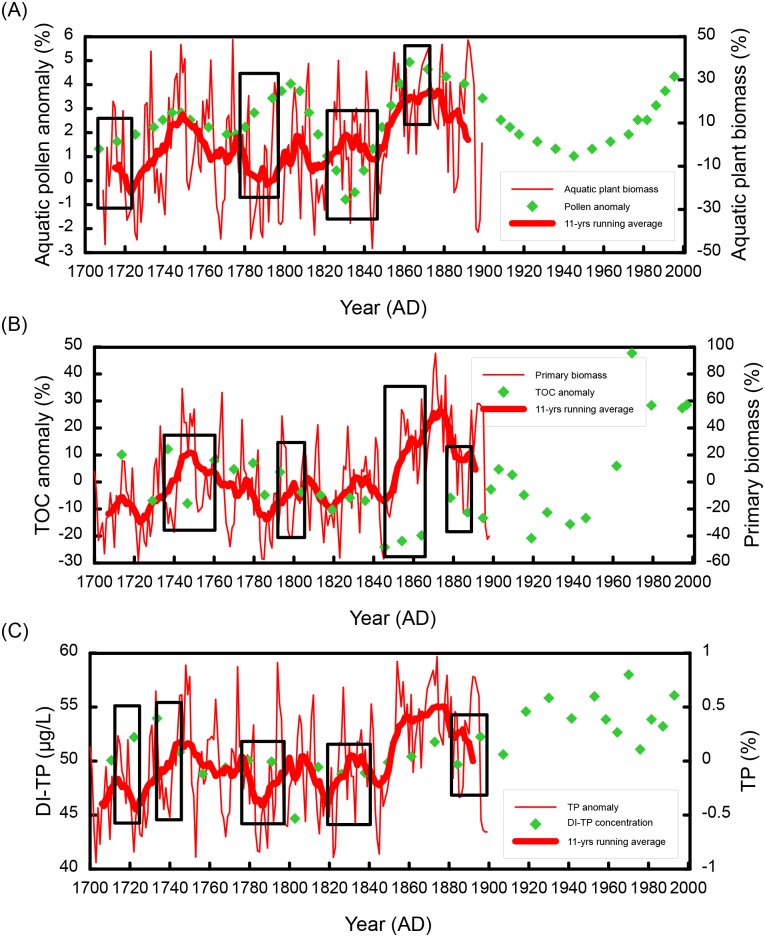
Validation of the Model 2 simulations (1700–1899). (A) Comparison between pollen concentration anomaly from core ZK01 [51] and simulated algae biomass anomaly; (B) Comparison between TOC anomaly from core DCH [36] and simulated primary biomass anomaly; (C) Comparison between DI-TP from core WC-2 and simulated TP anomaly.

The simulated biomass and TP anomalies were subsequently compared with the sediment records (Fig 5). Due to the coarse resolution of the sediment records, it is difficult and unreasonable to perform a quantitative correlation analysis between the sediment proxies and simulations. Consequently, we visually compared the simulation results (red line in Fig 5) and the proxy data (green symbol in Fig 5), then determined the periods of different (rectangular boxes in Fig 5) and similar trends. Subsequently, we estimated that approximately 60%~70% of the years from1700-1899 showed similar trends between the simulation results and the proxy data, indicating that the simulation results captured most of the variations of the sediment proxies. Therefore, we considered the simulation results reliable.

### Relationship between nutrient accumulation and HRT

**Fig 6:**
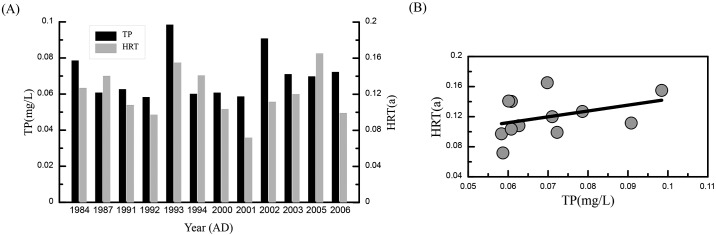
(A) Observed TP concentrations and the corresponding HRTs for 12 years from 1984–2006; (B) Correlation between the observed TP concentrations and the corresponding HRTs for these years.

To validate the relationship between nutrient accumulation and HRT, we compared the observed TP concentrations with the corresponding HRT values. The HRT data were continuous, while TP concentrations were not observed annually. Therefore, years for which both HRT data and observed TP concentrations were extant were selected for comparison (Fig 6A) and linear correlation analysis (Fig 6B). The analysis results showed a correlation coefficient of 0.685 (*p*<0.05), indicating a good positive linear relation between nutrient concentration and HRT.

## Discussion

The trophic state of a lake can generally be related to a gradual increase in nutrients, especially of phosphorus and nitrogen. This will lead to the abnormal growth of phytoplankton and aquatic plants, a decrease in the diversity and stability of the aquatic community, and deterioration in the water quality [90]. Lake ecosystems and their related hydrology are the primary constraints on a eutrophic state during lake trophic evolution. First, lakes are open systems in which materials and energy can freely exchange with the surrounding environment [91]. Both the primary producers and consumers in a lake ecosystem absorb nutrients during their lives, but release nutrients and organic material when they die [92]. Second, the water exchange rate significantly impacts nutrient accumulation processes. Nutrient accumulation and algae blooms occur more easily when a water body is isolated [93], resulting in a longer pollutant residence time. Therefore, the residence time of substances in a lake is a practical and powerful indicator of the nutrient supply and storage within the lake [92]. Many biological and hydrological processes are interactively linked in a lake ecosystem. These ecological phenomena normally vary within bounded ranges, but rapid and nonlinear responses can be triggered by the smallest changes if threshold values are exceeded [2,94].

### Response of lake nutrients to HRT

Eqs 2 and 3 indicate that a long HRT is the result of reduced runoff or effective precipitation, which causes a low water exchange rate and weak hydrodynamic intensity, and a slow flow velocity. More nutrients may be retained in a lake under such conditions. On the other hand, a short HRT corresponds to strong water exchange and hydrodynamic intensity. The nutrients in the water are more easily flushed from the lake. The correlation between the observed TP concentration and the HRT in Poyang Lake (Fig 6) validated this assumption and confirmed that the HRT affected the nutrient accumulation in the lake. Therefore, under natural conditions that are primarily governed by climate and hydrological factors, the HRT can be used as an index to evaluate the trophic state of Poyang Lake.

### Biomass response to lake nutrients

**Fig 7:**
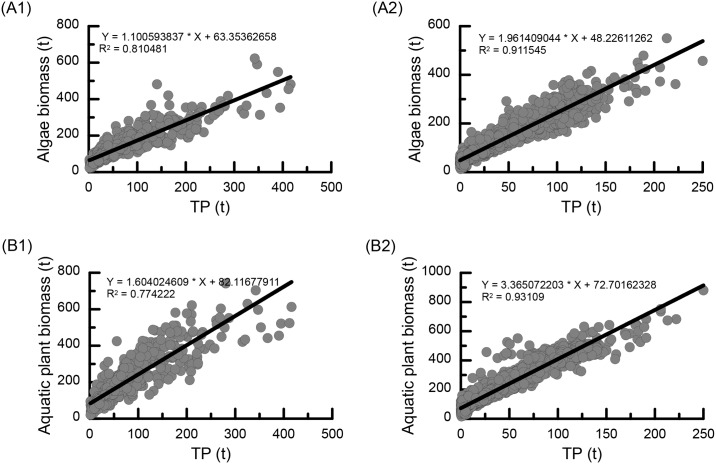
Correlations between the simulated algae biomass and the TP content for 1955–2008 (A1) and 1700–1899 (A2); Correlations between simulated aquatic biomass and TP for 1955–2008 (B1) and 1700–1899 (B2).

We did simple linear regression analyses with confidence interval of 95% using TP as the independent variable and the biomass as the dependent variable to illuminate the relationships between the algae and aquatic plant biomass and the water TP content in Poyang Lake. The F-test result showed that the regression equations were accurate. Additionally, the determination coefficients (R^2^) fell between 0.77~0.93 (Fig 7), implying that the water TP had a clearly positive effect on the changes of the algae and aquatic plant biomass.

**Fig 8:**
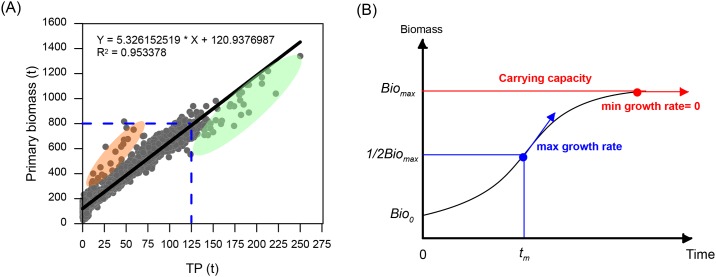
(A) Relationship between the simulated primary biomass and TP; (B) A S-shaped curve showing the growth pattern of the living organisms (redraw and modified from Rubin and Davidson, 2001, [95]).

However, a scatterplot of primary biomass (i.e., the total biomass of algae and aquatic plants) and the water TP content showed that more points occurred above the linear trend line (black solid line in Fig 8A) when TP< 125 t (red shadow in Fig 8A), while the most points fell below the line when TP> 125 t (green shadow in Fig 8A). This phenomenon can be explained by the S-shaped growth pattern shown in Fig 8B. This pattern can be described by a logistic function, first introduced by the Belgian mathematician Pierre-Francois Verhulst in 1838 [95]. According to the function, the living organism initially begins to grow exponentially, but with time, as the biomass reaches its half maximal value (expressed as 1/2Bio_max_ in Fig 8B), the growth rate (slope of the S-shaped curve) gradually slows, until it finally reaches zero (the slope shown as red arrow in Fig 8B). Before the biomass reaches 1/2Bio_max_, it is primarily controlled by food or the nutrient supply—for example, the water TP in this study. However, the environmental capacity subsequently becomes an important limiting factor of the biomass increase. The growth rate slows down even if resources persistently increase. In this study, the 1/2Bio_max_ was estimated as approximately 800 t in Poyang Lake, when the water TP was 125 t (blue dash line in Fig 8A). Therefore, it can be inferred that the algae and aquatic plant growth rate first increases and then declined after the biomass reaches 800 t. Additionally, TP = 125 t becomes a threshold for algae and aquatic plant growth, which indicates the inflection point between an increasing and decreasing growth rate.

### Identification of the driving forces

Because of the different driving forces and feedback in the two models, comparisons of the two simulations can help identify the dominant factors and elucidate the mechanisms behind the trophic changes in Poyang Lake. The HRT in Model 1 was largely affected by the effective precipitation (P-E) and runoff, so it can reflect trophic changes in the lake that are controlled by climatic and hydrological factors. TP in Model 2 is the synthetic result of the external climatic-hydrological forces and the internal biomass feedback. The standard deviation (SD) is a parameter that is used to quantify the amount of variation or the dispersion of a dataset. The deviation of the HRT series was caused by fluctuating climatic and hydrologic factors, while deviation in the TP series resulted from the volatility of both climatic-hydrological factors and the lake primary biomass. Therefore, we suggest that the SD can be used as a measurement of the contribution of climatic-hydrological factors and the lake primary biomass to the trophic changes in the lake.

**Fig 9:**
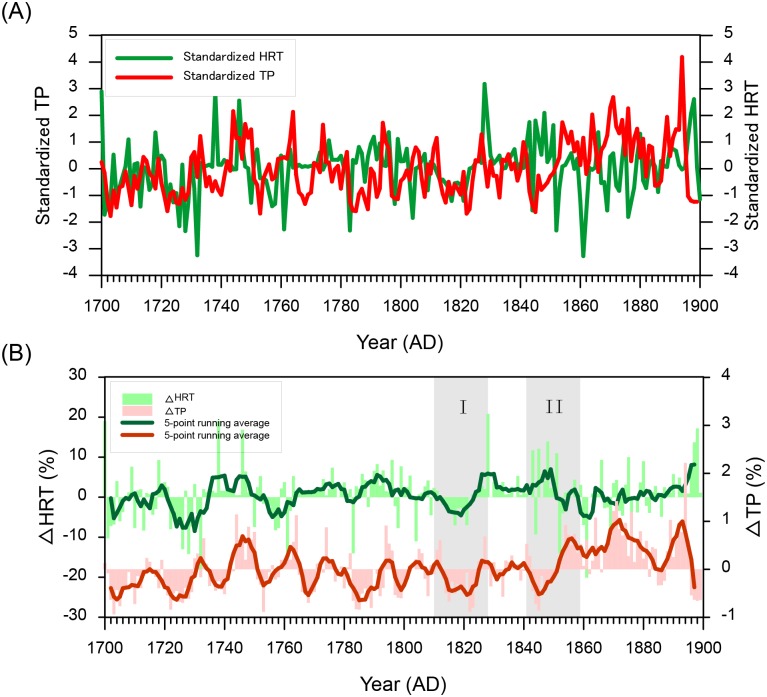
(A) Standardized HRT and simulated TP series from 1700–1899; (B) Comparison between ΔHRT and simulated TP from 1700–1899.

The HRT and TP series were standardized to eliminate the effect of their different units and dimensions (Fig 9A) before a comparison was made. The results of MK tests for the standardized HRT and TP series suggest that approximately 62.5% of the variations is consistent and approximately 12.5% is inverse, and the approximately remaining 25% is uncertain. The different change patterns between HRT and the simulated TP imply different contributions of the climatic-hydrological factors and primary biomass to eutrophication in the lake. To elucidate the driving mechanism behind eutrophication process in Poyang Lake, two typical periods, 1812–1828 (consistent-change stage, rectangle I in Fig 9B) and 1844–1860 (inverse-change stage, rectangle II in Fig 9B), were selected. During the consistent-change stage (1812–1828), the standard deviations of standardized HRT and TP series were 0.727 and 0.919, respectively, suggesting that HRT captured 79.1% of the variations in TP. The other 20.9% was attributed to primary biomass changes. However, in the inverse-change stage (1844–1860), the contribution of the primary biomass to the TP variations was estimated as 63.6% according to the SDs of the standardized data of HRT and the simulated TP.

Based on these analyses, we suggest that the primary biomass was the major contributor to the trophic changes in the lake during the inverse-change stage. But, during the consistent-change period, climatic-hydrological forces govern the trophic changes. We estimated that consistent changes were predominant in the period from 1700–1899 AD. Consequently, it can be inferred that water exchange in Poyang Lake plays a very important role in the trophic changes in the lake. Although inverse changes only accounted for 12.5% of the variations during this period, the primary biomass also appeared to be an important driving force in the long-term trophic change process.

## Conclusions

In this study, we used a hydraulic residence model and a lake ecology-nutrient model to simulate long-term trophic change process in Poyang Lake. The HRT was driven by climatic and hydrological forces, and was shown to be a practical index that reflects the trophic state of Poyang Lake. The simulated TP was controlled by the dual effects of climatic-hydrological forces and the primary biomass. The consistent and inverse changes between the HRT and simulated TP allow an insight into the driving mechanisms driving them. The climatic and hydrological factors were found to be the primary driving forces in most of the years we examined (1700–1899), which indicates that water exchange between Poyang Lake and the surrounding rivers is greatly important in preventing eutrophication. Regulatory authorities should not establish redundant water conservation facilities in Poyang Lake and its connected rivers that may restrict water exchange. The feedback between the primary biomass and lake nutrients (water TP in the present study) is also a factor that cannot be ignored. However, we found that the algae and aquatic plant growth rate declined after the biomass reached the half maximal value, and that the biomass increased almost linearly with an increasing TP content. This implies that water TP is absorbed by algae and aquatic plants for growth and reproduction. During an inverse-change period with a long hydraulic residence time (or high ΔHRT), more TP accumulated in Poyang Lake. However, the simulations revealed a lower TP content in the corresponding period, which made the feedback of algae and aquatic plants obvious during this time. Therefore, regulatory authorities should consider that optimizing the aquatic ecosystem structure may be effective for controlling nutrient.

Uncertain periods during the time series (i.e., neither definitely consistent nor inverse) indicated that other driving forces affect the trophic evolution process. Because a lake is an open system, many external factors such as human activity interfere with lake development and eutrophication. Exploring methods that can determine the main driving forces controlling trophic evolution in a lake is an important issue for future research.

## Supporting Information

S1 Fig Profile curves of ^137^Cs_ex_ and ^210^Pb_ex_, and the relationship between depth and age. (TIF)

S2 Fig Diatom percentage diagram for core WC-2, with taxa abundance >10%. (TIF)

S1 Table Ratio estimates of environmental resources of algae, aquatic plant and fish biomass in Poyang Lake. (DOC)
