# Supplementary figures and images for: Eutrophication in Poyang Lake (Eastern China) over the Last 300 Years in Response to Changes in Climate and Lake Biomass
Source: PLoS One. 2017 Jan 3;12(1):e0169319. doi: 10.1371/journal.pone.0169319 (PMC5207526; doi:10.1371/journal.pone.0169319)

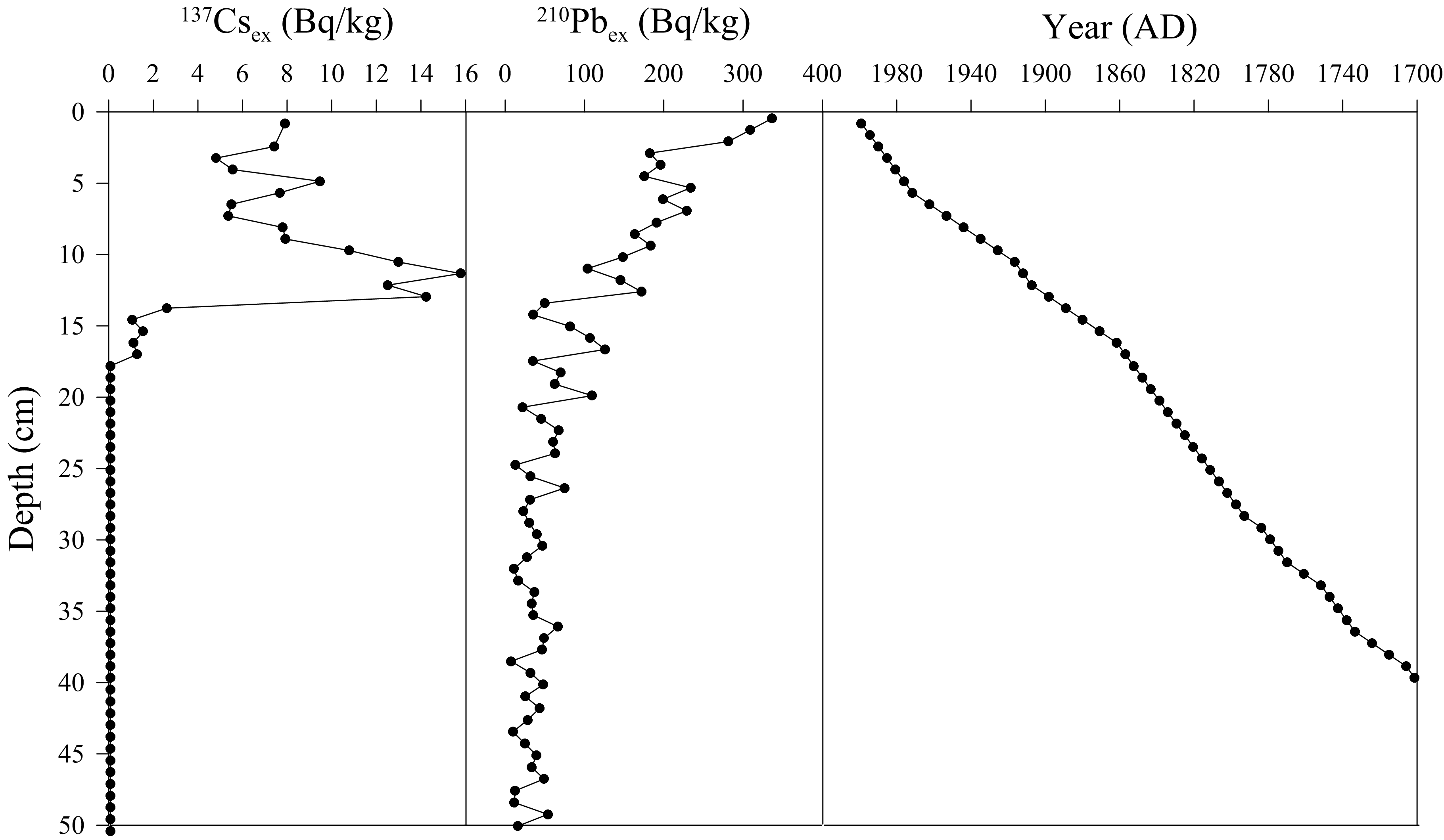

Supplement: S1 Fig — (TIF) [file pone.0169319.s001.tif]

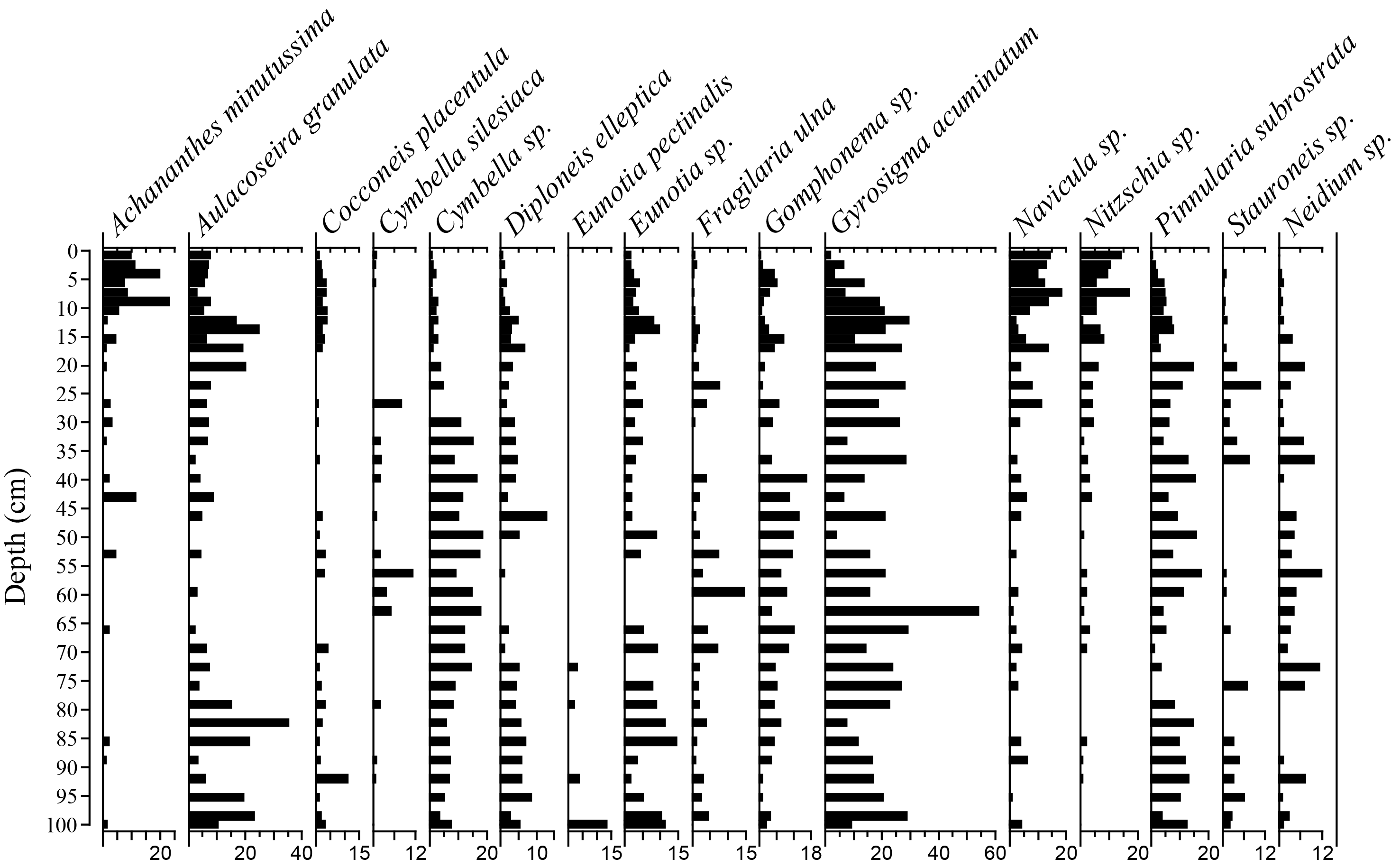

Supplement: S2 Fig — (TIF) [file pone.0169319.s002.tif]
