# Supplementary material for: Eutrophication in Poyang Lake (Eastern China) over the Last 300 Years in Response to Changes in Climate and Lake Biomass
Source: PLoS One. 2017 Jan 3;12(1):e0169319. doi: 10.1371/journal.pone.0169319 (PMC5207526; doi:10.1371/journal.pone.0169319)
Supplement: S1 Table — (DOC) [file pone.0169319.s003.doc]

S1_Table Ratio estimates of environmental resources of algae, aquatic plant and fish biomass in Poyang Lake

| Annual biomass  (×10^4^ t/a) | Phytoplankton | Aquatic plant | Fish  (estimated biomass) | Fish  (1950s maximal fish catches) | Fish  (1990s maximal fish catches) |
| --- | --- | --- | --- | --- | --- |
| Guan et al. (1987) |  | 451.33 |  |  |  |
| Jin et al. (1990) |  | 388 |  |  |  |
| Zhu et al. (1997) | 451.32 | 652.9 | 2.08 (1960s) | 1.360 | 4.4553 |
| Special Report of apply and protection for the fish resources of Poyang Lake (2011) | 451.33 | 652.9 |  | 2.158 | 7.190 |
| Zhou et al. (2011) |  |  | 4.4114  (2011) |  |  |
| Adoption | 451.33 | 652.9 |  |  | 4.4553 |
| Ratio | biomass algae: aquatic plant: fish ≈100:145:1 | | | | |
